# Extended-release tofacitinib for refractory Behçet disease: A case report

**DOI:** 10.1097/MD.0000000000029189

**Published:** 2022-04-15

**Authors:** Chrong-Reen Wang, Tak-Wah Wong, Sheng-Min Hsu

**Affiliations:** a Department of Internal Medicine, National Cheng Kung University Hospital, Tainan, Taiwan; b Department of Dermatology, National Cheng Kung University Hospital, Tainan, Taiwan; c Department of Ophthalmology, National Cheng Kung University Hospital, Tainan, Taiwan.

**Keywords:** Behçet disease, extended-release tofacitinib, JAK inhibitor, Th1/Th17-type cytokines

## Abstract

**Rationale:**

Although single-cytokine inhibitors can be considered in treating severe or refractory Behçet disease (BD), these biologic agents are associated with potential therapeutic failure due to the multi-cytokine pathogenesis involving Th1- and Th17-type cytokines with activated Janus kinase/signal transducer and activator of transcription signaling pathways. Notably, there is an increasing trend toward the use of small-molecule targeted drug tofacitinib (TOF), a pan-Janus kinase inhibitor, with immediate-release formulations for treating patients with severe or refractory systemic vasculitis involving different vessel sizes. Despite no reported efficacy of extended-release formulations in refractory BD yet, such a dosage form has pharmacokinetic parameters that are comparable to those of conventional immediate-release formulations.

**Patient concerns and diagnosis:**

We report the case of a 27-year-old local woman with recurrent manifestations of arthritis, orogential ulcerations, papulopustular lesions, and anterior uveitis. She was diagnosed with BD for more than 3 years, and received long-term corticosteroids plus immunosuppressants therapy with the complication of opportunistic candidiasis infection.

**Interventions and outcomes:**

Under extended-release TOF 11 mg once-daily therapy, the patient achieved disease remission while sparing the use of corticosteroids during follow-up.

**Lessons:**

Our clinical observations implicate the oral convenience and therapeutic efficacy of extended-release TOF formulations in controlling the disease activity of BD.

## 1 Introduction

Behçet disease (BD), first described by Hulusi Behçet from Istanbul in 1937, is an autoimmune systemic vasculitis affecting small and large vessels with widespread presentations including recurrent orogenital ulcerations and articular, cutaneous, gastrointestinal, neurological, ophthalmological, and vascular involvement.^[1]^ A better understanding of pathophysiological mechanisms and novel therapeutic options for clinical manifestations can improve the management of this disease and raise the quality of life in such patients. Biologic agents and small molecule-targeted drugs such as anti-tumor necrosis factor (TNF) monoclonal antibodies (mAbs) and apremilast have been recommended by the European League Against Rheumatism to treat severe or refractory disease.^[2]^ Tofacitinib (TOF), the first small-molecule pan-Janus kinase (JAK) inhibitor, was approved by the Federal Food and Drug Administration (FDA) in November 2012 for the treatment of rheumatoid arthritis (RA) with moderate to high activity and an inadequate response to methotrexate.^[3]^ Since individual cytokine receptors can recruit their own combinations of JAKs to activate distinct processes in targeted cells, antagonizing a specific JAK can inhibit more than 1 cytokine pathway, thereby expanding the therapeutic efficacy.^[4]^ Based on the distinct mechanisms of action, JAK inhibitors are at a focus of research in treating miscellaneous autoimmune and inflammatory disorders, and have the potential to provide positive outcomes by sparing the use or minimizing the dosages of corticosteroids (CS) and nonspecific immunosuppressants.^[5]^

Despite not an indicated use, there is an increasing trend toward using TOF with immediate-release formulations in difficult-to-treat systemic vasculitis involving different vessel sizes, including antineutrophil cytoplasmic antibody-associated vasculitis, BD, polyarteritis nodosa, and Takayasu arteritis (TAK).^[6–9]^ Notably, extended-release formulations have pharmacokinetic parameters, tolerability and safety that are comparable to those of conventional immediate-release formulations.^[10]^ Herein, we report a case of refractory BD with articular, mucocutaneous and ocular manifestations under long-term CS plus immunosuppressants therapy complicated by opportunistic candidiasis infection. She received extended-release TOF 11 mg once-daily therapy, leading to disease remission while sparing the use of CS.

## 2 Case presentation

**Figure 1:**
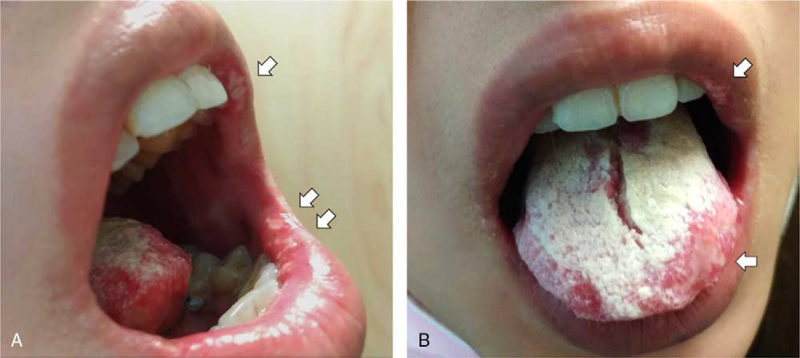
A. Multiple irregular-shaped ulcers over the lips (arrows). B. Whitish patches over the tongue with the isolation of *Candida glabrata*. Irregular-shaped ulcers over the lip and tongue (arrows).

A 27-year-old local woman was admitted to the Internal Medicine Department of the National Cheng Kung University Hospital with a 2-week history of orogenital ulcers, polyarthralgia and swallowing pain. Before her visit, she was diagnosed with BD at other hospitals for more than 3 years, based on the manifestations of arthritis, orogential ulcerations, papulopustular skin lesions, and uveitis.^[11]^ Furthermore, despite under a long-term prescription of CS plus colchicine and immunosuppressants such as methotrexate, there were recurrent articular, mucocutaneous and ocular activities. Except for BD, the patient had no other co-morbidities. At admission, physical examination revealed a body temperature of 37.5°C, injected eyes and multiple irregular-shaped ulcers over the tongue, gingiva, palate, and lips as well as whitish patches over the tongue, palate and throat (Fig. 1). There were swollen and/or tender joints over the wrist, knee, and foot areas. Gynecological consultation revealed multiple vulvar erosions with pimples. Laboratory profiles showed elevated CRP (15 mg/L) levels and ESR (28 mm/h), leukocytosis (12,300/L) with a dominant neutrophil classification (85%), and unremarkable liver/renal function and urine analysis. Hepatitis B, hepatitis C, and human immunodeficiency viral markers were absent. Autoantibody profiles including antinuclear antibody, antineutrophil cytoplasmic antibody and rheumatoid factor were negative. Cultures of tongue patches yielded *Candida glabrata*, whereas no microorganisms were isolated from the blood samples or vulvar lesions. There were no abnormalities on chest radiograph, electrocardiogram or abdominal echography. Intravenous injections of high-dose CS (1 mg/kg/d prednisolone equivalents) and oral fluconazole/nystatin were prescribed for disease activity and oropharyngeal candidiasis, respectively, with clinical improvements. After discharge, she underwent regular follow-up at the outpatient rheumatology clinic with an additional prescription of azathioprine (2 mg/kg/d). Nevertheless, a relapse of disease activity occurred with articular, mucocutaneous, and ocular manifestations during tapering down the daily CS dosages. Ophthalmological consultation revealed iritis without chorioretinitis, which was suggestive of anterior uveitis. Owing to the reported efficacy of TOF for refractory BD^[7]^ and patient's preference for once-daily therapy, we initiated extended-release TOF 11 mg once-daily treatment in June 2021. During follow-up, she had disease remission with normalized CRP levels, ESR and leukocyte counts. The daily use of prednisolone was discontinued with TOF monotherapy for more than 6 months.

## 3 Discussion

Cytokines produced by T helper (Th) cells, Th1 and Th17, can bind to types I and II cytokine receptors associated with JAKs, causing their phosphorylation and further recruitment of signal transducer and activator of transcription (STAT) proteins to modulate gene transcription in pathogenic cells involved in autoimmune diseases.^[12]^ It has been demonstrated that both Th1 and Th17 activities are upregulated in BD,^[13,14]^ similar to the T-cell responses in RA, a systemic rheumatic disease with the therapeutic indication of JAK inhibitors.^[15]^ The activation of JAK/STAT signaling pathways in BD is mediated through the interaction of Th1- and Th17-type cytokines with their individual surface receptors on the targeted cells.^[16,17]^ Although single-cytokine inhibitors including anti-TNF mAbs and interleukin (IL)-6 blockers can be considered in severe or refractory BD,^[18]^ these biologic agents are associated with potential therapeutic failure due to its multi-cytokine pathogenesis. Notably, TOF has been shown to treat autoimmune diseases by suppressing the differentiation of pathogenic Th1 and Th17 cells through the inhibition of T-bet and STAT1 activation.^[19]^ Interestingly, owing to the multi-cytokine mechanism involving Th1 and Th17 pathways, therapeutic failure under anti-TNF or anti-IL-6 therapy has been observed in TAK patients resistant to conventional disease-modifying anti-rheumatic drugs.^[8]^ In 13 patients with refractory TAK including 11 treated earlier with biologic agents, 10 had clinical benefits after receiving TOF therapy for 2 to 18 months.^[20]^

A recent study in BD examined the efficacy of immediate-release TOF 5 mg twice-daily therapy under preserved background medications in 13 patients with articular, cardiovascular, and/or gastrointestinal manifestations refractory to conventional disease modifying anti-rheumatic drugs.^[7]^ Among them, 5 cases had ever received anti-TNF mAbs and 1 was treated earlier with an IL-6 blocker. Their disease activities improved after a follow-up period of 5 to 21 months, with remission in articular and cardiovascular manifestations. The lack of effects on their gastrointestinal involvement might be attributed to the resemblance of such a manifestation in BD to Crohn disease for which TOF is known to be ineffective.^[21]^ Moreover, another report investigated 13 patients with severe posterior uveitis including 6 refractory to anti-TNF therapy.^[22]^ They received 30 mg daily doses of prednisone and 5 mg TOF 2 times a day, resulting in a rapid and sustained improvement in visual acuity. Despite no cardiovascular, gastrointestinal involvement and posterior uveitis in our reported patient, extended-release TOF 11 mg once-daily therapy led to disease remission with weaning off the daily use of CS.

Although there are no available data demonstrating the superiority of TOF over biologic agents in treating refractory BD patients, using this small-molecule targeted drug is associated with oral convenience and therapeutic efficacy as demonstrated in previous studies and this reported case.^[7,22]^ On the basis of the equivalence in the area under the plasma concentration-time curve between the 2 formulations with evidence from the exposure-response relationship that area under the plasma concentration-time curve is the relevant parameter for clinical responses,^[23]^ extended-release TOF formulations with osmotic drug delivery were granted regulatory approval by the FDA for RA therapy in February 2016 without the need for advanced clinical trials.^[24]^ Later on, the FDA approved new indications for such formulations in treating patients with active diseases of psoriatic arthritis, ulcerative colitis and ankylosing spondylitis in December 2017, December 2019 and December 2021, respectively. Despite not an indicated use, extended-release TOF has been shown to have therapeutic effects that are not inferior to immediate-release formulations in refractory TAK.^[20]^ In addition, extended-release formulations enable less frequent dosing than immediate-release formulations. Collectively, these findings suggest that extended-release TOF can provide the less frequent oral dosing convenience with similar efficacy in comparison with immediate-release formulations in treating refractory systemic rheumatic diseases.

## 4 Conclusions

There is an increasing trend toward the use of TOF with immediate-release formulations in refractory systemic vasculitis involving different vessel sizes. We report the case of 27-year-old local woman with BD. She had recurrent articular, mucocutaneous and ocular manifestations, and received long-term CS plus immunosuppressants therapy complicated by the opportunistic candidiasis infection. Under extended-release TOF therapy, the patient achieved disease remission with sparing the use of CS. Our clinical observations implicate the oral convenience and therapeutic efficacy of extended-release TOF formulations in controlling the disease activity of BD.

## Acknowledgments

The authors are indebted to the doctors and nurses involved in the management of the reported case at the National Cheng Kung University Hospital.

## Author contributions

**Conceptualization:** Chrong-Reen Wang, Tak-Wah Wong.

**Data curation:** Chrong-Reen Wang, Tak-Wah Wong.

**Formal analysis:** Chrong-Reen Wang, Tak-Wah Wong, Sheng-Min Hsu.

**Investigation:** Chrong-Reen Wang, Tak-Wah Wong, Sheng-Min Hsu.

**Supervision:** Chrong-Reen Wang.

**Validation:** Chrong-Reen Wang, Tak-Wah Wong, Sheng-Min Hsu.

**Writing – original draft:** Chrong-Reen Wang.
